# Isoenergetic-practical and semi-purified diets for protein requirement determination in *Hermetia illucens* larvae: consequences on life history traits

**DOI:** 10.1186/s40104-021-00659-y

**Published:** 2022-01-19

**Authors:** Sara Bellezza Oddon, Ilaria Biasato, Laura Gasco

**Affiliations:** Department of Agricultural, Forest and Food Sciences, University of Turin, Largo P. Braccini 2, 10095 Grugliasco, TO Italy

**Keywords:** *Hermetia illucens*, Life history traits, Nutritional requirements, Semi-purified

## Abstract

**Background:**

Black soldier fly (BSF) is one of the most promising species for the intensive breeding of insects given its adaptability and its efficiency in the conversion of waste. To maximize the production and use waste as substrates, it is essential to determine the larvae nutritional requirements. The study aims to evaluate the effects of 5 practical, semi-purified and isoenergetic diets (PSPID) with increasing protein levels (10%, CP10; 14%, CP14; 16%, CP16; 19%, CP19) on BSF life history traits. A total of 2000 six-day-old larvae were weighed and divided into groups of 100 (4 replicates/treatment [PSPID and Gainesville diet (GA) used as environmental control]). In larva-prepupa stage, sampling was carried out on 30 individuals/replicate, while in prepupa-adult stage on 35.

**Results:**

In the whole larval stage, the CP16 treatment performed better when compared to the other groups. At 18 days old, the CP14 treatment showed a higher weight than the CP19 (*P* < 0.01), while the CP10 and CP16 weights were intermediate. On the contrary, the CP10 prepupae weight was higher than the CP19 (*P* < 0.01). The CP10 and CP14 pupae performed better in terms of weight when compared to the others (*P* < 0.01). The dietary treatments did not affect the adult parameters. The sex significantly influenced both the exuvia weight, which was the greatest in females (*P* < 0.001), and the fly lifespan, longest in males (*P* < 0.05). Fly lifespan was also influenced by the interaction between treatment and sex, with the CP19 females showing a longer life than the others (*P* < 0.05).

**Conclusions:**

In conclusion, the optimal protein level considering the whole larval stage is 16% on dry matter (DM). At 18 days old, looking at the size intended for the meal production, it can be considered 14% on DM. The result obtained on adult emergence in PSPID may not be attributable to the protein content. Further researches on macronutrients requirements determination have to be conducted to evaluate the substrates composition effects on BSF life history traits.

## Background

In recent years, the number of studies and commercial development related to the insect production for recycling, reutilization and reuse of waste biomass from agri-foood system has significantly grown [1, 2]. In Europe, the insect species that can currently be bred for fish, poultry and pig feed production are as follows: the black soldier fly (*Hermetia illucens*), the housefly (*Musca domestica*), the yellow mealworm (*Tenebrio molitor*), the lesser mealworm (*Alphitobius diaperinus*) and three different species of cricket (*Acheta domesticus*, *Gryllodes sigillatus, Gryllus assimilis*) (Reg. UE 2021/1372). Among them, the black soldier fly (BSF) is one of the most promising for the intensive breeding, given its adaptability, its efficiency in the conversion of waste, and the simple management of its life cycle compared to the other insect species [3].

According to scientific publications, the black soldier fly larvae can be fed a wide range of organic materials [4] such as restaurant waste [5], vegetable and fruit waste [6], rice straw [7], poultry manure and human faeces [8].

The BSF breeding is focused not only on the re-utilization of waste materials as feed resources, but also on the maximization of the production (which is the primary goal of the livestock farming). However, these two aspects partially contrast with each other. Indeed, the maximization of the production is generally achieved with a standardized process that is difficult to obtain using only waste as substrates, since they are characterized by a strong seasonality and linkage with the territory. In fact, the produced raw materials (food/feed destination) have an environmental vocation and, consequently, the same applies for the resulting waste. However, unlike the raw materials, in order to respect the circular economy model, the displacement of waste is incoherent.

Therefore, the gap between these two aims needs to be reduced in order to create a sustainable insect farm. To achieve this, it is fundamental to determine the nutritional requirements in larval stage and, in turn, formulate diets that maximize the production using waste. The scientific literature concerning the assessment of BSF larvae dietary macro-element requirements is still very limited. In the last few years, researchers have mainly focused on the evaluation of the effects derived from the interaction of macro-elements, for example protein (P) and carbohydrate (C). In particular, Barragan-Fonseca et al. [9] have formulated diets with different P:C ratio (10:35, 10:45, 10:55, 17:35, 17:45, 17:55, 24:35, 24:45, 24:55, respectively) containing chicken feed in a fixed amount and different quantities of starch, casein and cellulose. Furthermore, Cammack and Tomberlin [10] took into consideration not only the P:C ratio (7:35, 21:21 and 35:7, respectively), but also the association with different moisture percentages. Both the studies revealed that diets containing high amount of carbohydrate increased the development time in black soldier fly larvae. Different protein levels in substrates composed by food by-products have also been reported to influence the BSF larval development, with the fastest growth being observed on the high protein and fat diets (CP: 21.9% of the dry matter [DM]; EE: 9.5% DM) [11]. Furthermore, the larval yield and individual pupal weight were the greatest in the diet with the highest carbohydrate content for all the three protein levels (10%, 17% and 24%) [9].

Apart from investigating the relationship between the different macro-elements, it is also necessary to determine the optimal levels of each individual nutrient for the BSF larvae. The currently available information in other animal species could lay the foundations for pilot studies in insects. Indeed, nutritional needs of the animal species used for zootechnical or experimental purposes have usually been determined by using purified or semi-purified diet [12–15]. The purified diet is a feed made out of refined ingredients with precisely defined composition (i.e, starch and casein), while the semi-purified diet contains some natural ingredients in a relatively pure form (nearly 100%) in combination with purified ingredients [16]. Finally, another type of experimental diet to test new ingredients in feed is the practical diet, which is formulated from natural ingredients as cereal grains, oil seed meals and fish meal [16].

Based on the above-reported background, the present study aims to evaluate the effects of different practical, semi-purified and isoenergetic diets (PSPID) with increasing protein levels on larval development and mortality, and adult parameters.

## Material and method

### Experimental diets

In the trial, five different experimental diets were tested: four practical semi-purified and isoenergetic diets with increasing protein levels (10%, CP10; 14%, CP14; 16%, CP16; 19%, CP19), and the standard Gainesville diet (GA) used as environmental control (Table 1). To maintain experimental diets isoenergetic, a reduction of lipid was performed by decreasing the corn inclusion. The diets were considered practical and semi-purified since they consist of raw materials derived from cereal production (i.e. corn and rice husk) and semi-purified ingredients (casein and starch). The GA was composed by corn, wheat bran and alfalfa as indicated by Hogsette [17]. The GA treatment was considered as the environmental control in order to exclude the possible effects of both the microclimatic alterations and the handling procedures on the obtained results.

**Table 1:** Ingredients (g/kg as is), chemical composition (g/100 g as is, unless otherwise stated), and gross energy (MJ/kg as is) of the five experimental diets

Items	CP10	CP14	CP16	CP19	GA
Ingredients, g/kg					
Whole rice husk	100	100	100	100	–
Chopped rice husk	237.5	311.4	374.2	410.2	–
Starch	300	230	170	135	–
Casein	62.5	96.6	130.8	164.8	–
Corn	300	262	225	190	200
Alfalfa	–	–	–	–	300
Wheat bran	–	–	–	–	500
Total	1000	1000	1000	1000	1000
Chemical composition^a^					
DM	87.69	89.40	89.74	90.22	88.30
CP as is	9.97	13.95	16.54	19.42	13.55
EE as is	1.27	1.07	0.98	0.92	2.22
Ash as is	5.00	6.40	7.48	8.29	5.79
aNDFom as is	23.77	25.77	33.81	32.05	34.91
NSC^1^ as is	49.13	55.78	67.27	68.98	67.00
GE, MJ/kg as is	15.95	16.03	16.20	16.32	16.09

Legend: *CP10* 10% CP diet, *CP14* 14% CP diet, *CP16* 16% CP diet, *CP19* 19% CP diet, *GA* Gainesville diet. *DM* dry matter, *CP* crude protein, *EE* ether extract, *GE* gross energy, *aNDFom* amylase neutral detergent fiber organic matter, *NSC* non-structural carbohydrates.

^a^Values are reported as mean of duplicate analyses

^1^Calculated as 100 - [(100 - DM) + CP + EE + Ash + aNDFom]

As a first activity, the corn was ground to 2 mm diameter by cutter (CL/5 Fimar, Italy) before being mixed with the semi-purified components (casein and starch), and the obtained mixture was kept at 4 °C. Before being used, the mixture was acclimated for 4 h at 28 °C. Just before the actual usage, the whole rice husk and 116.6 g of water (T = 28 °C) to reach the 70% of moisture were added to the meal ingredients (50 g). The whole rice husk (10% of DM) was included in the formulation and added before the substrate use to prevent an excess substrate cohesiveness. A sample of each substrate was frozen at − 20 °C for the chemical analysis.

### Chemical analyses

To properly formulate isoenergetic diets with increasing protein levels, before the diet formulation all the raw materials were chemically analysed. In particular, the dry matter (DM; AOAC #934.01), the crude protein (CP; AOAC #984.13; conversion factor N × 6.25) and the ash (AOAC #942.05) were determined by the International AOAC [18], the ether extract (EE; AOAC #2003.05) by the International AOAC [19] and the amylase neutral detergent fiber organic matter (aNDFom) by Mertens [20]. The NSC was calculated using the following formula:

$$ NSC=100-\left[\left(100-\mathrm{DM}\right)+\mathrm{CP}+\mathrm{EE}+\mathrm{Ash}+\mathrm{aNDFom}\right]. $$

The gross energy was analysed using an adiabatic calorimetric bomb (C7000; IKA, Staufen, Germany). Moreover, after the substrate preparation, a sample of each dietary treatment was analysed with the same procedures listed above to control the accuracy of the substrate formulation.

### Larval stage

The experiment was performed using the BSF colony established at the Experimental Facility of the Department of Agricultural, Forest and Food Sciences (University of Turin). Collected BSF eggs were incubated and the 1-day-old larvae were harvested and fed the GA until the beginning of the trial.

A total of 2000 six-day-old larvae were weighed (pool of 10 larvae per single weight; initial larvae weight: 0.07 g ± 0.02) with a precision balance (Kern & Sohn GmbH; Balingen, Germany; d = 0.001), randomly divided into groups of 100 and allocated in 20 boxes (10 cm × 17.5 cm × 7 cm) covered by a perforated cup (4 replicates per dietary treatment). Each box was filled with 50 g of substrates (0.5 g/larva) that underwent an acclimatization period of 1 h in the climatic chamber in order to avoid a thermal shock for the larvae. The boxes were placed in the climatic chamber with controlled condition (T°: 28 ± 0.5 °C; RH: 70 ± 5%; 16:8 L:D). Every day, the boxes were checked and the substrate was gently mixed with a spoon to prevent the casein surfacing (superficial sedimentation). Throughout the trial, 50 g of feed was added in all the replicates (each treatment received 0.08 g/larva/day) every 4 d until the prepupae appearance (18 days of age). After the beginning of the trial, a total of three samplings (30 larvae/replica) at 4-day intervals were carried out when larvae were 10 (T1), 14 (T2) and 18 (T3) days old to evaluate the growth. Before weight recording, the larvae were cleaned with warm tap water (28 °C) and gently dried with a paper. As the procedure was not destructive, once weighted, the larvae were returned to the box. At the end of the experiment, when 40% of the prepupae were identified, the total number of larvae was counted for the survival calculation.

### Prepupal stage

Once the appearance of the first prepupae, no substrate was furtherly added to the boxes. An inspection was made every day to count the prepupae. When 40% of the prepupae was identified, the larval development was considered ended and a sampling was carried out (30 prepupae/replica) to evaluate their weight.

### Pupal stage

In comparison with the prepupa, the pupa is characterized by a rigid puparium and the absence of elasticity [21]. After being identified, the pupae weight was recorded and the larva-pupa phase duration (L-P) was determined (35 individuals/replica). A total of 35 pupae per replica were positioned in perforated plastic boxes (10 cm × 8 cm × 8 cm; 1 pupae/box) and placed inside the climatic chamber (T°: 28 ± 0.5 °C; RH: 70 ± 5%; 16:8 L:D). To assess the time for the adult emergence, the pupae check was carried out twice a day.

### Adult stage

The days needed for the adult emergence for each pupa (pupa-fly phase duration [P-F]) were recorded. Once fly emerged, the sex of the fly and the weight (fly live weight [FLW]) were recorded. The flies were not fed during their lifetime.

In order to record the FLW, the containment boxes were individually weighted. The FLW was then calculated as the difference between the weight of the box with the fly and the exuvia, and the weight of the empty box and the exuvia recorded at the fly death. The flies were kept in the same climatic chamber of the larvae, prepupae and pupae. At the death, the dead fly weight (DFW) and the exuvia weight (EW) were recorded. The DFW was used for the calculation of the weight reduction (WR) percentage between the FLW and DFW. Moreover, the fly life span (FLS, days) was also evaluated. In absence of fly emergence, after 1 week since the death of the last fly per each experimental treatment (minimum time: 3 weeks, maximum time: 1 month), the pupae were declared dead and the emergence rate (ER) was calculated.

$$ ER=\frac{n{}^{\circ} emerged\ flies\times 100}{n{}^{\circ} pupae\ collected\  per\  treatment} $$

The effect of the sex on adult stage parameters was finally assessed.

### Development score

**Fig. 1:**
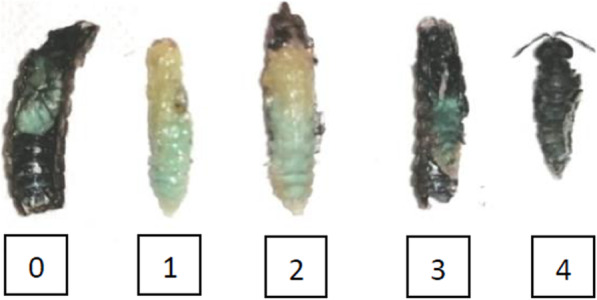
Photographic-development stage score of the non-emerged flies. 0 = absence of formation, 1 = anatomical formation of the fly without pigmentation, 2 = presence of the fly with pigmented eyes, 3 = fly with the pigmented thorax, 4 = presence of an anatomically complete fly

With the aim of setting up a development score, before the beginning of the trial, a total of 200 pupae in different intra-puparial stage were dissected in order to evaluate their development status. On the basis of this preliminary study (data not shown), in addition to the research carried out by Barros-Cordeiro et al. [22], a photographic-development score has been fine-tuned. A score from 0 to 4 was assigned based on the characteristics of the fly (Fig. 1). The 0 corresponded to the absence of formation, 1 to the anatomical formation of the fly without pigmentation, 2 to the presence of the fly with pigmented eyes, 3 to the fly with the pigmented thorax, and 4 to the presence of an anatomically complete fly. At the end of the trial, all the dead pupae were dissected to determine the development stage of the fly.

### Statistical analysis

The statistical analysis was performed using the IBM SPSS Statistics software (V27.0.0). The normality or non-normality distribution of the collected data was determined by Shapiro-Wilk test. The experimental unit for all the analysed parameters for the larval, prepupal, pupal and adult stages was the box. On the contrary, the fly represented the experimental unit for the adult ER. Data related to the GA substrate were excluded from the statistical analysis as it was considered as the environmental control only. The larval weights were analysed by fitting a generalized linear mixed model that allowed them to depend on two fixed effects (diet and time [plus, accordingly, their interaction]) through a gamma probability distribution with a nonlinear link function (log). The replicate was included as a random effect to account for repeated measurements on the same box. The interactions between the levels of the fixed factors were evaluated by means of pairwise contrasts.

One-way ANOVA (post-hoc test: Tukey) was, instead, used to compare the L-P phase and the larval survival among the experimental diets. Differently, prepupae and pupae data were tested by Kruskal-Wallis test (post-hoc test: Dunn’s Multiple Comparison Test). Finally, the adult stage data were analysed by fitting either a general or generalized linear mixed model, with the only exception of the ER being evaluated with the one-way ANOVA test (post-hoc test: Tukey). In particular, a general linear mixed model allowed the FLW and the EW to depend on two fixed effects (diet and sex [plus, accordingly, their interaction]), while a generalized linear mixed model allowed the WL, P-F duration and FLS to depend on the same fixed effects through a gamma probability distribution with a nonlinear link function (log). In both the statistical models, the replicate was included as a random effect to account for repeated measurements on the same box, and the interactions between the levels of the fixed factors were evaluated by means of pairwise contrasts. A likelihood-ratio test was also performed in case of not significant interaction terms, and, when necessary, a model simplification was applied by removing them from the statistical models. The results were expressed as mean (L-P phase, larval survival, prepupal and pupal weights, and ER) or least square mean (larval weight, FLW, WL, EW, P-F duration and FLS) and pooled standard error of the mean (SEM). The level of significance considered was < 0.05.

## Results

### Larval stage

Data recorded for larvae grown on GA diet were consistent with those usually recorded in the experimental facility (data not shown) and allowed to exclude any environmental issues during the trial. At the beginning of the trial, the larvae weight was homogeneous in all the dietary treatments (0.070 ± 0.001; *P* > 0.05). Considering the whole larval stage (Table 2), the CP16 and the CP14 treatments were significantly different (0.170 and 0.163, respectively; *P* < 0.001). On the contrary, the weight of the CP19 larvae was intermediate, being comparable to the experimental diets with 14% and 16% of protein (*P* > 0.05). The CP10 displayed a lower weight when compared to the other dietary treatments (*P* < 0.001). The sampling time influenced the weight of the larvae, as it reflected the physiological larval growth (*P* < 0.001).

**Table 2:** Effects of diet, time and interaction between diet and time on larvae weight (1 larva/weight)

Parameter	Diet (D)				Time (T)			SEM		***P***-value		
	CP10	CP14	CP16	CP19	10 d	14 d	18 d	D	T	D	T	DxT
Weight	0.148^c^	0.163^b^	0.170^a^	0.168^ab^	0.083^c^	0.221^b^	0.231^a^	0.004	0.001	0.001	0.001	0.001

Legend: *CP10* 10% CP diet, *CP14* 14% CP diet, *CP16* 16% CP diet, *CP19* 19% CP diet, *GA* Gainesville diet, *d* days old, *SEM* standard error of the mean.

Means with different superscript letters (a, b, c) within the same row differ significantly (*P* < 0.05)

**Fig. 2:**
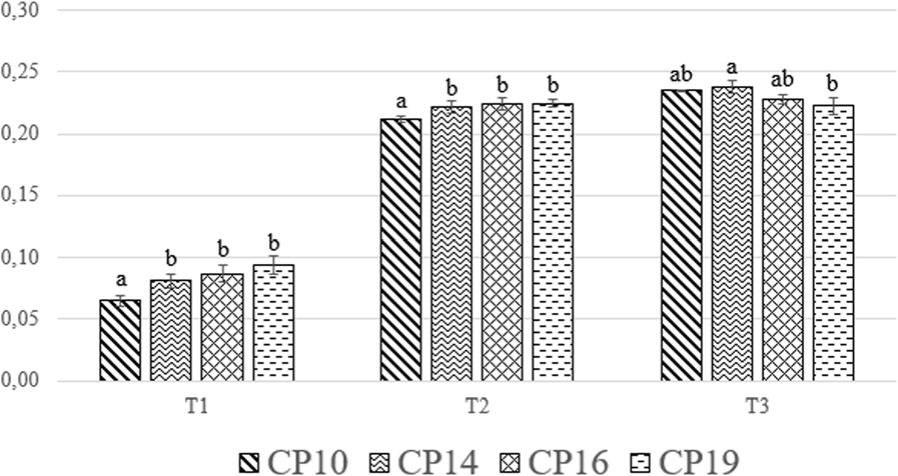
Larvae growth performance. Graph bars with different superscript (a, b) indicate significant differences among the experimental diets within the sampling time (*P* < 0.05)

Figure 2 shows the interaction between diet and time. The CP14, CP16 and CP19 treatments showed improved growth at 10 and 14 days old (T1 and T2, respectively) in comparison with the CP10 (*P* < 0.001). At 18 days old (T3), the CP19 group showed a lower weight when compared to CP14 larvae (0.223 and 0.238, respectively; *P* < 0.01). The CP10 and the CP16 groups did not differ from the other dietary treatments.

As regards the survival rate, no differences were observed among the experimental diets (CP 10–14-16: 89% and CP19: 90%, *P* > 0.05).

### Prepupal stage

**Fig. 3:**
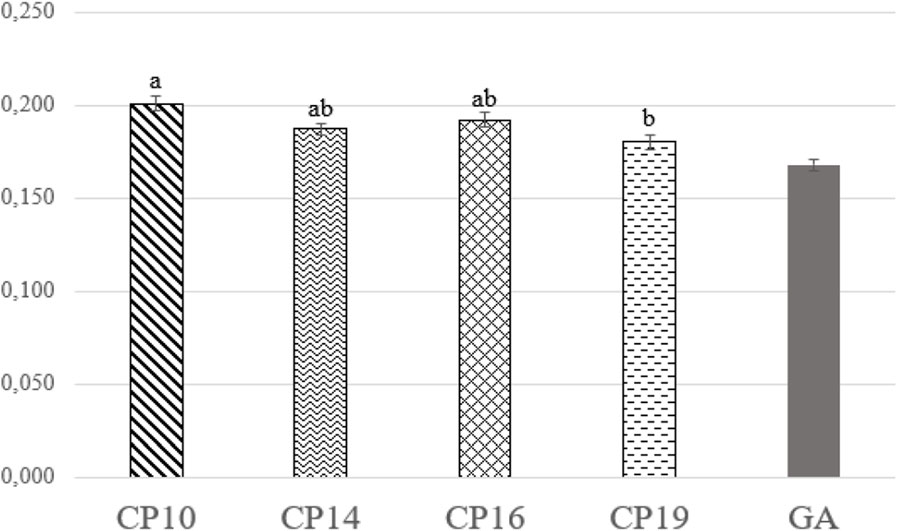
Prepupae weight (1 prepupa/weight). Graphbars with different superscript letters (a, b) differ significantly (*P* < 0.05)

Figure 3 summarizes the prepupae weight. In particular, the CP10 treatment was statistically different from the CP19 group (*P* < 0.01). On the contrary, the CP14 and the CP10 prepupae weight was intermediate between the lowest and the highest protein level (*P* > 0.05). The GA prepupae appeared less heavy than those of the experimental diets.

### Pupal stage

Table 3 shows the effects of the dietary treatments on pupae weight, L-P phase duration and pupae score. In the pupal stage, the CP10 and CP14 treatments showed heavier pupae when compared to the CP16 and CP19 (0.188–0.194 and 0.172–0.174, respectively; *P* < 0.001). The CP16 and CP19 pupae had a similar weight (0.172 and 0.174; *P* > 0.05). On the contrary, the environmental control (GA) displayed the numerically lowest weight.

**Table 3:** Effects of the dietary treatments on pupae weight, larva-pupal phase duration and pupae score

Parameters	Dietary treatments				SEM	***P*** -value	GA
	CP10	CP14	CP16	CP19			
Weight, g	0.188^a^	0.194^a^	0.172^b^	0.174^b^	0.004	0.000	0.154
L-P duration, d	21.11^bc^	21.80^a^	20.88^c^	21.57^ab^	0.104	0.000	16.43
Score	3.98	3.98	3.95	3.99	0.206	0.206	3.80

Legend: *L-P* larva-pupal phase duration (n° of days), *CP10* 10% CP diet, *CP14* 14% CP diet, *CP16* 16% CP diet, *CP19* 19% CP diet, *GA* Gainesville diet.

Means with different superscript letters (a, b, c) within the same row differ significantly (*P* < 0.05)

As regards the L-P phase duration, the larvae that took the longest to pupate were the CP14 (21.80 d), while the shortest time was observed for the CP16 (20.88; *P* < 0.001). The timing of the CP19 treatment was comparable to the CP10 and CP14 groups (*P* > 0.05), while it was statistically different from the CP16 treatment (*P* < 0.001). The GA insects showed a L-P duration time of 16.43 d, thus being apparently shorter than the ones reported in the PSPID.

All the development scores applied to the moribund pupae were close to 4 and no significant differences between the dietary treatments were recorded (*P* > 0.05).

### Adult stage

**Fig. 4:**
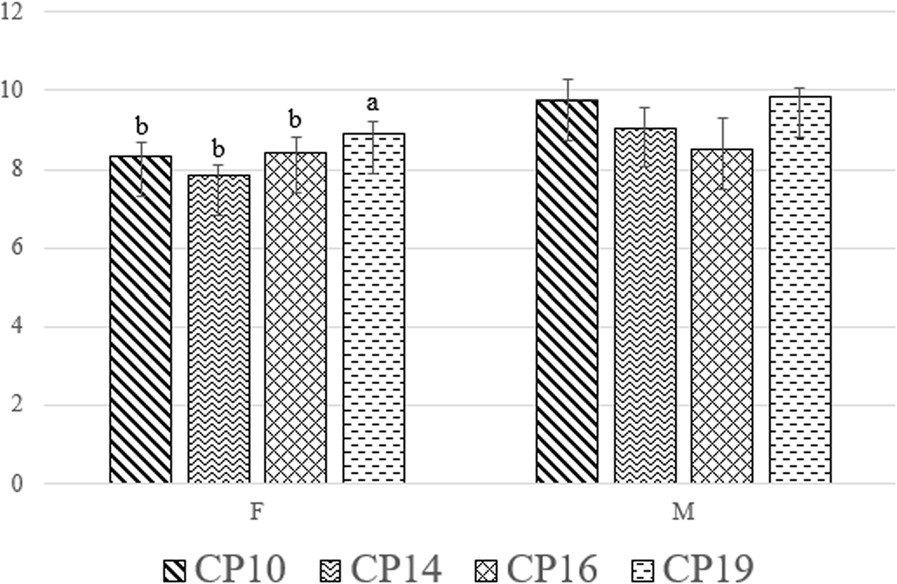
Fly lifespan. Graph bars with different superscript (a, b) indicate significant differences among the sex within the dietary treatments (*P* < 0.05)

The effects of the dietary treatments and sex on the adult stage parameters are reported in Tables 4 and 5. No differences among the diets were reported for FLW, WR and EW (*P* > 0.05, Table 4). On the contrary, the sex influenced the weight of the exuvia, which was greater in the females than the males (*P* < 0.01, Table 4). The P-F duration time was not affected by the dietary treatments, the sex and the interaction between the two factors (*P* > 0.05; Table 5). Fly sex had an effect on the FLS (*P* < 0.05, Table 5). In particular, the males lived almost a day longer than the females (*P* < 0.01). The CP19 female flies also showed a longer lifespan when compared to the females from the other groups (*P* < 0.01, Fig. 4). Regarding ER, no differences were observed among the experimental diets (*P* > 0.05). Moreover, the ER displayed by flies reared on experimental diets were very low when compared to the ones reared on the GA diet, despite being in the same environmental conditions (CP10 37.1%, CP14 35.7%, CP16 25%, CP19 35% and GA 86,4%).

**Table 4:** Effects of diet and sex on the fly live weight, fly weight reduction and exuvia weight

Parameters	Diet (D)				Sex (S)		SEM		***P***-value	
	CP10	CP14	CP16	CP19	F	M	D	S	D	S
FLW	0.126	0.139	0.134	0.124	0.140	0.121	0.010	0.007	0.647	0.076
WR	48.87	48.16	49.27	50.24	47.34	48.86	3.031	1.246	0.155	0.068
EW	0.023	0.023	0.024	0.024	0.025	0.022	0.001	0.001	0.303	0.001

Legend: *CP10* 10% CP diet, *CP14* 14% CP diet, *CP16* 16% CP diet, *CP19* 19% CP diet, *GA* Gainesville diet, *FLW* fly live weight, *WR* fly weight reduction, *EW* exuvia weight, *SEM* standard error of the mean

**Table 5:** Effects of diet, sex and interaction between diet and sex on pupa-fly duration time and fly lifespan

Parameters	Diet (D)				Sex (S)		SEM		***P***-value		
	CP10	CP14	CP16	CP19	F	M	D	S	D	S	D x S
P-F	7.04	7.02	7.29	6.72	7.19	6.84	0.281	0.218	0.090	0.248	0.365
FLS	9.01	8.43	8.45	9.36	8.36	9.27	0.342	0.314	0.211	0.035	0.001

Legend: *CP10* 10% CP substrate, *CP14* 14% CP substrate, *CP16* 16% CP substrate, *CP19* 19% CP substrate, *GA* Gainesville diet, *P-F* pupa-fly duration time, *FLS* fly lifespan, *SEM* standard error of the mean

Means with different superscript letters (a, b, c) within the same row differ significantly (*P* < 0.05).

### Gainesville diet

Data regarding the larva, pupa and adult parameters recorded for the GA are summarized in Table 6.

**Table 6:** Larva, pupa and adult parameters recorded for the environmental control

Larvae (day old)	Weight, g
6	0.070
10	0.089
14	0.204
18	0.167
**Pupae**	
Weight, g	0.154
L-P duration, d	16.43
Score	3.80
**Adult**	
WR, %	49.51
FLW, g	0.103
EW, g	0.019
P-F duration, d	7.55
FLS, d	8.10

Legend: *L-P* larva-pupal phase duration, WR fly weight reduction, FLW fly live weight, EW exuvia weight, *P-F* pupa-fly duration time, *FLS* fly lifespan

## Discussion

### Larval stage

To the author’s knowledge, the present study is the first to evaluate the effects of isoenergetic diets with increasing levels of protein on the BSF larva development and flies-related parameters. The BSF is one of the most promising insect species for feed production given its ability to convert waste [3]. However, determining the protein requirement can improve its efficiency and guarantee the optimization of the waste reduction. Handling has been reported to affect either the weight or the survival of the larvae [23]. However, since all the treatments in this experimental trial have undergone the same handling procedures, the impact derived from the manipulation of the larvae can be reliably excluded. The data related to the GA treatment fell within the typical ranges for each development stage, thus making possible to state that no environmental effects altered the herein obtained results. During the whole larval development, the protein level that gave the best result in terms of growth was the 16% on the DM. This result is in agreement with that obtained by Lalander et al. [8] on the larvae reared on poultry feed with a protein content of 17.3% DM, but not with the weight of those raised on primary sludge containing the 16.9% CP on DM. In particular, the average weight of the larvae at 18–20 days of age grown on the poultry feed lied in a range between 200 to 250 mg per larva, while that of the larvae grown on the primary sludge was in 100–150 mg range [8]. Considering the limited protein variation between the two diets, it is presumed that the difference in weight is not due to the protein content, but to the other macronutrients.

At 10 and 14 days of age, the CP10 larvae showed the lowest weight when compared to the other experimental treatments. The CP10 larvae weight (10 days old) was in agreement with that recorded by Meneguz et al. [6] on the vegetables and fruit (VEGFRU) substrate with 11.9% DM of protein level (about 60 mg/larva). On the contrary, at 14 days old, the CP10 larvae weight was almost twice that was recorded on the VEGFRU by the same authors. Considering 18-day-old larvae, the protein level that gave the best result in terms of weight was the one containing 14% of protein on DM (238 mg/larva). Differently, Lalander et al. [8] observed the lowest larvae growth (0–50 mg/larva) in a substrate with a protein level of 14.7% on DM. The results heterogeneity herein reported may be related to the type of supplied protein or the substrate texture. Since experimental diets with higher protein levels performed better in the first two growth periods, while the opposite trend was observed in the third one, it is possible to state that the protein level can be reduced in the last stages of larval development without affecting the growth performance. This condition, from a production point of view, allows the use of waste with a lower protein content in instars close to the prepupae. Given the reduced weight gain between 14 and 18 days of age, this moment can be considered the plateau phase. As a consequence, in terms of meal production, the 14th day of larvae life could be considered optimal. To reach this development stage with the best performance it is recommended to use protein levels equal to or greater than 14% on DM.

The larval survival rate was high in every treatment, being also greater than that observed by Oonincx et al. [11] in diets with different C:P ratios (72–86%) and Cammack et al. [10] in P:C balanced diet (57–62%). On the contrary, the rate registered in the present study was in agreement with that of Chia et al. [23] on brewing-waste reared larvae. A reduced mortality rate means that the compositions of all diets met the minimum larval nutrient and physical requirements.

### Prepupal stage

The weight of the prepupae ranged from 0.180 g to 0.201 g was globally higher than that recorded in Cammack et al. [10] study (range: 0.080–0.110 g). Furthermore, in their study, the 70% humidity control diet performed better than the others with different C:P ratios and moisture content [10]. This result is in contrast to what was found in the present study, in which the GA control (30% of DM) showed the lowest weights. The data obtained from the prepupae of the CP16 treatment was slightly less than those of the Lalander et al. [8] research, where the BSF larvae were fed poultry feed-based diet (0.251 g). The prepupae of primary sludge treatment, as well as the larvae, showed a different weight when related to the results of the present study. The CP16 prepupae performed better even compared to the weight of the prepupae obtained by Spranghers et al. [5] on restaurant waste with a CP percentage of 15.7%. Since similar protein levels were not accompanied by similar results, it may be argued that the diversity of raw materials used in the experimental diets have a major influence, and that semi-purified ingredients would have been more easily metabolized and, consequently, assimilated. The CP10 treatment reported the highest values, with statistical differences against the CP19 diet. In the prepupae phase, the macronutrient *par excellence* that guarantees the reserve for the non-feeding phase is the lipid. For this reason, in the last stages of the mobile period, a reduction in the protein content may not cause any negative consequences on BSF growth.

### Pupal stage

In the pupal stage, even in the last instar larvae and prepupae, the best performance in terms of weight were observed in the experimental diets with lower protein levels. Considering the time needed for larvae to reach pupae stage (L-P duration), all the experimental treatments showed values close or higher than 21 d, while GA larvae took about 16.5 d. It has been reported that the larvae development time is usually affected by the quantity of macronutrients in the diets, specifically lipids [24]. If the substrate is low in fat, the larvae need more time to acquire their fat body and, consequently, take longer to complete their growth [24]. This last statement agreed with our results, since the experimental diets were characterized by a lipid content that varied from 0.92 to 1.27% (as is), while the GA showed an EE value of 2.22% (as is). The absence of isolipidic diets may represent a limitation of the present study. However, at the same time, this gap represents the importance of determining the minimum macronutrient requirements in order to guarantee improved performance in all the phases of the BSF life cycle.

### Adult stage

Data related to the life parameters may have been influenced by the rearing box. In particular, the flight space was reduced compared to the cages one. Moreover, it is possible to hypothesize a reduced energy consumption compared to the breeding conditions. Only the sex influenced the size of the flies and the exuvia weight. Specifically, the males tended to be smaller than the females, thus being in agreement with Jones and Tomberlin [25], which observed that, in their colony, the weight of the males was constantly lower when compared to the females. The EW displayed the same results in relation to the sex, as it was significantly greater in the females than the males. The WR was not affected by the dietary treatments, but it showed a tendency to significance in relation to the sex as well. In particular, the males tended to show higher weight loss due to the longer lifespan when compared to females. Furthermore, females have been reported to display a highest quantity of dry matter, thus reliably explaining the reduced weight loss [26]. Gao et al. [27] also observed a longer lifespan in males than females (10.32 and 9.31 d, respectively). The females accumulate higher energy reserve than males for the eggs production [28]. Since in the present trial the mating was not possible and the females lived less than the males, it is possible to speculate that the energy reserve accumulated for reproductive purposes – in absence of reproduction activity – was not channelled for other uses.

The females of the diet with the highest protein level lived longer than the others, thus making reasonable to hypothesize that the protein accumulated in the larval stage, unlike the energy, may have an effect on the adult life duration.

The total duration in days of the period between the pupal stage and the adult emergence was studied by Barros-Cordeiro et al. [22]. These authors demonstrated that under controlled conditions (27 ± 1.0 °C, 60 ± 10% RH, 12:12 L:D), the P-F duration is 8 d. In the present study, the numerically shortest period (6.7 d) was recorded in the experimental treatment with the highest protein level (CP19). However, the differences between the treatments that took less time for the adult emergence and that took the longest (CP16) did not exceed 1 d. The difference in the results obtained from the two studies may derive from the adoption of different temperature and photoperiod conditions. Indeed, an increase in the temperature of the pupal mass can cause a pupal phase reduction, while an inadequate photoperiod can lengthen it [29].

The emergence rate was found to be extremely low in all the experimental groups. Considering that the dissected moribund pupae were at the most advanced development stage, it is possible to speculate that the reduced lipid intake of the practical semi-purified diets allowed the development of the fly in the puparium, but it was not sufficient to guarantee its emergence. Indeed, the experimental diets contained almost half of the lipid content (in percentage) compared to GA (CP10 was 43% less, CP14 52%, CP16 56% and CP19 58%). In Diptera, an eclosion hormone has been reported to influence the adult emergence [30]. Generally, the arthropod hormones involved in moulting and in other processes belong to the steroid class and are defined ecdysteroids [31]. Since insects cannot synthesize cholesterol de novo from acetate, they are dependent on the cholesterol ingested during the nutritional phase [31]. For this reason, the lipid component has another important function in the emergence moment. In particular, the fly must have the force to open the operculum of the puparium and crawl out of it [30]. Moreover, in nature the black soldier fly prefers the underground as pupariation site, and, consequently, it needs to dig the way upward through the soil [30]. Based on the above-reported argument, it is possible to hypothesize that, if the fly does not have an optimal quick-use lipid reserve, emerging may be hampered. This condition implies that, although a substrate guarantees good performance in the larval stage, it may generate disorders in the other phases of the life cycle. For this reason, the determination of the larvae nutritional requirements and the evaluation of the effects in prepupae, pupae and adults are necessary to formulate waste-based diets that do not lead to a deficit within the whole production cycle.

## Conclusion

The successful development of the BSF larvae in the PSPID demonstrates that they can be used for the determination of the nutritional requirements. Based on the results herein obtained, the optimal protein level for the maximum larvae size in the entire larvae stage might be considered 16% on DM, while at 18 days old the 14% on DM. Given the results obtained in the 18-day-old larvae, in the prepupa and pupa stages, it will be interesting to evaluate the possibility of using substrates with a lower protein content in the last development phases. The low adult emergence rate in PSPID diets may not be attributable to the protein, thus making the conduction of further researches mandatory in order to determine which nutrient deficiency leads to this outcome. Finally, this work lays the foundation for research in the evaluation of substrate qualitative assessment not only during the larval stage, but also in all BSF life history traits.

## Abbreviations

BSF: Black soldier fly
P: Protein
C: Carbohydrate
GA: Gainesville diet
DM: Dry matter
CP: Crude protein
EE: Ether extract
GE: Gross energy
aNDFom: Amylase neutral detergent fiber organic matter
NSC: Non-structural carbohydrates
SEM: Standard error of the mean
L-P: Larva-pupal
FLW: Fly live weight
DFW: Dead fly weight
EW: Exuvia weight
P-F: Pupa-fly
FLS: Fly lifespan
ER: Emergence rate
